# A Novel Complete-Surface-Finding Algorithm for Online Surface Scanning with Limited View Sensors

**DOI:** 10.3390/s21227692

**Published:** 2021-11-19

**Authors:** Alastair Poole, Mark Sutcliffe, Gareth Pierce, Anthony Gachagan

**Affiliations:** 1 Centre of Ultrasonic Engineering (CUE), University of Strathclyde, Glasgow G1 1XW, UK; s.g.pierce@strath.ac.uk (G.P.); a.gachagan@strath.ac.uk (A.G.); 2 The Welding Institute (TWI) Wales, Port Talbot SA13 1SB, UK; mark.sutcliffe@twi.co.uk

**Keywords:** NDT, free-form surface profiling, autonomous robotic systems

## Abstract

Robotised Non-Destructive Testing (NDT) has revolutionised the field, increasing the speed of repetitive scanning procedures and ability to reach hazardous environments. Application of robot-assisted NDT within specific industries such as remanufacturing and Aersopace, in which parts are regularly moulded and susceptible to non-critical deformation has however presented drawbacks. In these cases, digital models for robotic path planning are not always available or accurate. Cutting edge methods to counter the limited flexibility of robots require an initial pre-scan using camera-based systems in order to build a CAD model for path planning. This paper has sought to create a novel algorithm that enables robot-assisted ultrasonic testing of unknown surfaces within a single pass. Key to the impact of this article is the enabled autonomous profiling with sensors whose aperture is several orders of magnitude smaller than the target surface, for surfaces of any scale. Potential applications of the algorithm presented include autonomous drone and crawler inspections of large, complex, unknown environments in addition to situations where traditional metrological profiling equipment is not practical, such as in confined spaces. In simulation, the proposed algorithm has completely mapped significantly curved and complex shapes by utilising only local information, outputting a traditional raster pattern when curvature is present only in a single direction. In practical demonstrations, both curved and non-simple surfaces were fully mapped with no required operator intervention. The core limitations of the algorithm in practical cases is the effective range of the applied sensor, and as a stand-alone method it lacks the required knowledge of the environment to prevent collisions. However, since the approach has met success in fully scanning non-obstructive but still significantly complex surfaces, the objectives of this paper have been met. Future work will focus on low-accuracy environmental sensing capabilities to tackle the challenges faced. The method has been designed to allow single-pass scans for Conformable Wedge Probe UT scanning, but may be applied to any surface scans in the case the sensor aperture is significantly smaller than the part.

## 1. Introduction

Enabling robotised scanning processes is the harnessing of prior knowledge to fully traverse surfaces. For mobile or static-base robots completing NDT scans, knowledge of positions that have not been scanned is essential to ensure completeness of an inspection process that guarantees component integrity. Currently, this is ensured by planning a path over a known surface or part, that is then either verified of modified by an operator to ensure completeness.

Paths for parts equipped with an accurate CAD model can be produced automatically with commercial software. For parts without an accurate digital-twin, such as legacy parts or components with moulding errors, an operator has had to define a path on the robot’s teach-pendant manually to capture its unique profile.

For one-off scans or for scanning parts with unique moulding errors, this process voids the high speed and repeatability benefits available to robotised NDT. In these cases, robotic platforms must be able to flexibly scan parts through online path planning, and to provide the same guarantee of completeness in surface coverage that is achieved by a human operator manually inspecting the part.

Recently, NDT has been enabled to define a 2-scan process. The first scan reconstructs the part for path planning of a subsequent scan with NDT equipment. The second scan can then commence, fully covering the known surface that is within reach of the robot. Methods of reconstructing part surfaces in the initial scan have been widely researched with respect to both Photogrammetry and in the field of machining.

In the field of Photogrammetry, automated robotised methods for free-form surface profiling have developed significantly. Processes involving 3D or 2D cameras have evolved from requiring user-inputted positions [1] to fully automated 3D model reconstruction techniques. Automated photogrammetry has been applied to a wide range of scales, from fine-detail model reconstruction using robotic arms [2,3] to large-scale reconstruction using autonomous robots with wide-aperture sensors [4]. A recent example of photogrammetry enabling a 2-pass scan within NDT utilising Structure-from-Motion (SfM) [5].

These methods have relied on multiple volumetric inspections of a complex object using wide field-of-view sensors such as traditional RGB or RGB + Depth (RGB/D) cameras. This work has considered surface profiling in the case of limited-range sensors, such as line-scanners or ultrasonic devices that have a field of view many magnitudes smaller than the inspected surfaces. In the case of laser scanners, a volumetric pre-scan is not safe for human operators working nearby. Volumetric scanning of curved objects cannot guarantee surface discovery in the case of water-coupled ultrasound devices without lengthy re-scanning processes due to beam divergence and scattering.

Within the field of machining, validation of machining quality or accurate part profiling when there is no available CAD model has been implemented using Coordinate Measuring Machines (CMMs). CMMs utilising limited field-of-view sensors for full-surface profiling have also been thoroughly investigated [6]. Their use has relied on spline-surface approximations to predict surface positions [7,8,9], or planar raster-tangent path planning [10]. These methods all require saturation of user-sampled positions, user input to define surface tangents, or rely on tangents defined by a gantry constrained rasterization pattern. The spline-surface approximation method has been successfully applied to ultrasonic-sensor surface discovery [11]. This method requires that the surface can be defined by a global spline, as opposed to an atlas of piece-wise smooth splines. This is disadvantaged when inspecting objects with discontinuities such as holes, as these cannot be captured by a global b-spline representation. Surfaces with global b-spline representations are also known as doubly ruled surfaces.

In aiding accurate offline path planning for Eddy-Current inspections, CMM machinery and software were applied within a manual pre-scan procedure to generate a CAD model [12].

This work has sought to completely remove the reliance on operator inputted information regarding the target surface, except for its maximal curvature. The authors have further aimed to completely automate the surface-profiling process, unconstrained by sensor type, robotic platform, or spline representations of the surface. The only requirement on sensor information is that the position of the surface relative to the sensor and the normal-direction of the surface are recoverable at each scan position. Approximate normal direction extraction requires discovery of at least 3 accurate local surface points.

Enabling full surface discovery requires a search process and memory structure to discover and store potential surface points for later traversal.

**Figure 1:**
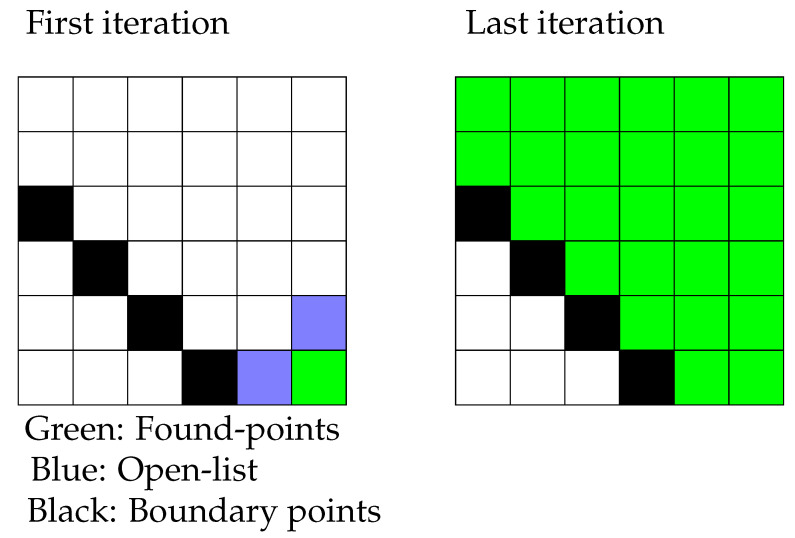
Colour Flood-Fill on the plane.

A candidate heuristic process are Flood Fill Algorithms (FFAs) that propagate through maps or networks in order to discover all positions within a connected surface or graph. The pseudo-code for two dimensional pixel maps has been presented in Algorithm 1 and accompanied by Figure 1.
**Algorithm 1** Flood Fill algorithm on the plane.1: FFA on the plane2: Begin at Pixel P_1_3: *Open*-*List* = {*P*_1_};4: *Points*-*Found* = {};5: **while** |*Open*-*List*| > 0: **do**6:  *P_a_* = *Open*-*List*.*back*()7:  *Points*-*Found*.insert(*P_a_*);8:  *Open*-*List*.delete(*P_a_*);9:  **for**
*direction* ∈ {′*UP*′, ′*DOWN*′, ′*LEFT*′, ′*RIGHT*′} **do**10:   *P_b_* = *P_a_* + *direction*11:   **if**
*P_b_* is new point AND not boundary point **then**12:    *Open*-*List*.*insert*(*P_b_*);13:   **end if**14:  **end for**15: **end while**

This work has generalised planar FFA heuristics to three-dimensional surface traversal, inventing the Complete-Surface Finding Algorithm (CSFA). Whereas FFAs require a pre-known data structure, the novel CSFA requires only curvature information about the target surface to ensure complete coverage when applied to sensors of arbitrary dimensions and sensitivity.

Simple stack-based FFA and scanline heuristics are of particular interest in the simulation section. Scanline implementations choose a preferred direction of motion for search until a boundary position is reached. When a boundary position is discovered, the less-preferable step is then taken until a free path is found in the preferred direction of motion. The resultant path is a traditional rasterization pattern, which is widely utilised within NDT path planning operations.

FFAs have been applied in various contexts, due to their simplicity and versatility. In the context of image processing, FFAs have seen ongoing widespread use in commercial products as a time-efficient method for filling a bounded region with a given colour [13]. The principle of the bucket-fill programme has been inverted to aid segmentation algorithms in 2D and 3D contexts from a user-inputted mask [14,15,16]. In recent years FFAs have aided machine-learning programmes in object recognition through automatic mask generation [17]. Mixed mapping and network theoretic implementations have been implemented to guide image reconstruction. First, FFAs were shown to be as effective as quality guided algorithms [18], and subsequently used to enhance nearest neighbour node quality optimisation methods in various fields [19,20,21].

Further, FFA variants have been extensively implemented in robotic path planning and control. Discretised potential field variants such as modified CFill and Flood-Field Methods (FFMs) have been shown to have greater time efficiency in comparison to Potential Field Methods (PFMs) [22,23]. FFAs have gained interest in the context of optimal path planning for 2D platforms [24,25], that has demonstrated flexibility through effective integration with optimal motion planners such as the A* algorithm [26]. These concepts have evolved in application to optimal motion planning in 3D space for UAVs with an exhaustive search pattern [27]. Further FFA integration and heuristic mirroring has shown to enhance traditional path planning algorithms [28,29]. The above Flood-Fill methods have been implemented on data either with a pre-defined link structure or with a full exploration in each potential direction. For unknown surface profiling constrained by costly rearrangement procedures and a limited field of view, these procedures are either non-applicable or significantly sub-optimal.

## 2. Method

The aim of this paper has been to generate a complete set of points that describe the full surface by utilising the simple operations presented in Algorithm 1. To embed planar FFA operations within a 3D context requires the local position and normal direction information at each position.

A point source has been placed with a given stand-off from the surface in the normal direction, and a ray is then generated to intersect with the surface from which the tangent directions have been extracted. The 3D analogue of moving in the 2D principle directions is given by approximating the local surface covered by the sensor array with a tangent plane, defined by the observed points and approximate normal direction. Given a surface normal, the principal axes corresponding to `UP’ and `DOWN’ directions have been calculated through the Gram–Schmidt orthonormalization process [30]. Given a normal vector $\vec{n}=\left[n_{x},n_{y},n_{z}\right]=\left[n_{i}\right]$, and principle directions ${\vec{e}}_{1}=\left[1,0,0\right]$, ${\vec{e}}_{2}=\left[0,1,0\right]$ and ${\vec{e}}_{3}=\left[0,0,1\right]$, the smallest component $\vec{x}$ has been selected as basis direction;

(1) $$\vec{x}=\left\{{\vec{e}}_{i}\mathrm{if}\left|n[i]\right|=\min_{k\in [1,2,3]}\left|n[k]\right|\right\}.$$

**Figure 2:**
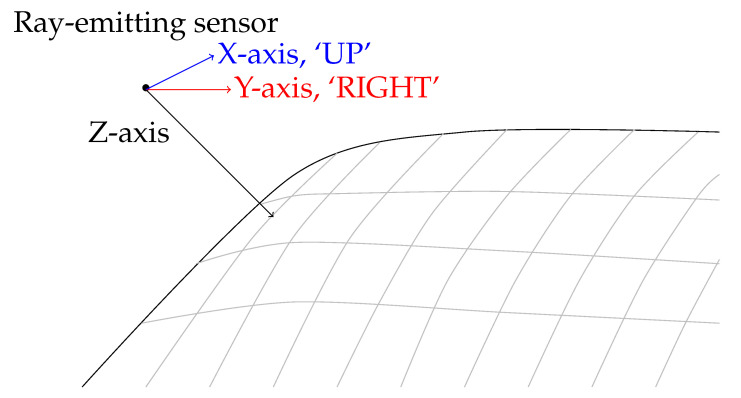
Flood Fill analogue in three dimensions. Grey lines represent iso-lines on the surface.

The chosen basis direction has then been orthonormalised with the surface normal through the Gram–Schmidt process. The next basis direction $\vec{y}$ is taken by cross product of normal and tangent vectors. The basis directions $\left[\vec{x},\vec{y}\right]$ have formed the cardinal directions that planar FFA’s utilise of ‘DOWN’ and ‘RIGHT’. The point source traverses the surface in an analogue implementation of the traditional planar FFA, displayed in Figure 2. If no data or insufficient data is available at a given position, the current search point is marked as being in the ambient space with no additional points hypothesised, representing the 3D analogue of a 2D boundary position.

The approximate local surface normal direction can be extracted from at least three distance measurements from a single position with a 2D sensor array. Well-calibrated 1D linear sensors arrays would require two measurement values within a small displacement range, and single-element 0D sensors would require data from at least three positions. The algorithm may be applied to any sensor capable of a surface-tool stand off measurement.

The authors have further adapted the simple embedded stack-based FFA implementation to produce a scanline variation that generates automatic rasterization patterns within post-processing. For surfaces with uni-directional curvature, this has been achieved by retaining the order of the extrapolated X,Y basis directions. Retaining order on surfaces with significant curvature in two directions, such as the sphere or bowl requires including a `preferable direction’ reference. This is so that when *X* and *Y* surface–tangent directions change their order during traversal, preference is given to the one that lies within a consistent plane in 3D space. On these surfaces, an irregular rasterization pattern emerges without preference vector. Irregular rasterization is not necessarily a negative feature, since for many robots and applications, there is an axial movement limit imposed that prevents multiple circular passes. This has been demonstrated in the results section, while rasterization is achieved in post-processing, online searches will require additional search positions that do not observe the target object in order to define boundary positions.

Finally a continuous surface must be discretised to ensure program closure, requiring a 3D analogue to 2D pixels. This structure allows positions that have been checked to be logged as seen. An Octree structure composed as a collection of boxes, or leaves has been chosen as it is less susceptible to numerical point-collisions present with a hash-table structure [31].

In order to assure full surface discovery, it is required that a step determined by the local information moves to a different Octree-node on the surface. Movements in 3D space under a set of changing basis directions may not align to a granular space oriented to the standard X,Y,Z bases. The undesirable effect of stepping within the same leaf may be prevented by moderating the Octree-leaf widths relative to the operator-specified step size *d*.

To ensure that each step defines a new leaf, the maximum potential length step within a leaf must be less than or equal to the step size. For leaf width *w* and step size *d*, the maximum step size, along the leaf’s diagonal can be restricted with Equation (2).

(2) $$w\leq \frac{d}{\sqrt{3}}.$$

On high-curvature surface sections the surface will inflect within each Octree leaf, reducing the Cartesian arc-length from one observed position to another. An upper bound for the arc-length reduction for curved surfaces needs to be defined to ensure that each step along the surface defines a new leaf.

Arc-length reduction due to the projection of a line along a curved surface is bounded by the surface’s curvature, which defines how a local linearisation deviates from the true surface profile. This term has been defined for a small step-vector $\vec{dx}$ by the Second Fundamental Form (SFF) denoted II [32];

(3) $$\mathrm{Arc}-\mathrm{length difference}\approx {\vec{dx}}^{T}\;\mathrm{II}\;\vec{dx}/2.$$

The principal curvatures of the surface are eigenvalues of the SFF, and so the maximum possible inflection of a curve bound to the surface is in the direction of maximum principal curvature.

If the maximal principle curvature over the surface is $\kappa_{\max}$, then an upper bound on the minimal required leaf-width for a step size *d* may be derived;

(4) $$w/d\leq \frac{\left|1-\left|\kappa_{\max}\right|d/2\right|}{\sqrt{3}}.$$

Dynamic discrete sampling may apply this principle to calculate minimal necessary Octree leaf-widths and step sizes in highly curved regions [33]; however, in this paper we restrict the analysis to uniform leaf-widths.

Flat surfaces have an over-sampling value of $w=d/\sqrt{3}$ (in units of *d*), since the maximal principal curvature is 0. This has returned Equation (2), since the step-size in ambient space is equivalent to that of the surface projection, the step taken always contained within the same spatial plane. An example of detrimental point-aliasing when curvature is not considered has been presented in the results section.

Finally, in the case of surfaces with a significantly restricted width, the step size should be limited to less than half of the minimum surface width.

The complete algorithm when simultaneously considering a pulse-echo test has been described in pseudo-code in Algorithm 2.
**Algorithm 2** Pseudo-code for the novel CSFA.1: Input: Maximum expected curvature *ĸ*, step-size *d*, and maximum Cartesian reach ∆*X*,2: Octree = GenerateWorkSpace(*ĸ*, *d*, ∆*X*),3: Operator moves sensor to surface,4: GetData() →surface position and normal vector *P*1, *N*1,5: *Open*-*List* = {P_1_}6: *Points*-*Found* = {}7: **while** |*Open*-*List*| > 0 **do**8:  P_a_ = *Open*-*List*.*back*()9:  *Open*-*List*.delete(P_a_)10:    **if** 0 < |*J_a_*^Ω^ {= InverseKin(*P_a_*)}| **then**11:   Move to *J_a_* = min_motion_
*J_a_*^Ω^12:   GetData() → *P_a_*, *N_a_*, data13:   **if** !data.*empty*() **then**14:    Sensor.z_*direction*_ → *N_a_*,15:    GetUTdata(),16:    *Octree*.insert(P_a_)17:    GramSchmidt(*N_a_*) → {′*UP*′, ′*DOWN*′, ′*LEFT*′, ′*RIGHT*′}18:    **for**
*direction* ∈ {′*UP*′, ′*DOWN*′, ′*LEFT*′, ′*RIGHT*′} **do**19:     *P_b_* = *P_a_* + *direction*20:     **if**
*P_b_* ∉ Octree **then**21:      *Open*-*List*.*insert*(*P_b_*);22:     **end if**23:    **end for**24:   **end if**25:  **end if**26: **end while**

**Figure 3:**
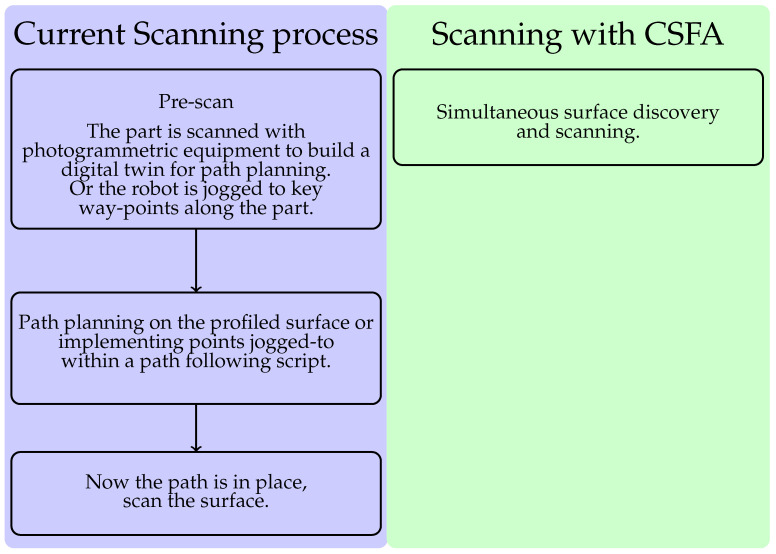
The one-step process enabled by the CSFA removes the necessity of accurate digital-twins and world-frame calibration, or lengthy robotic jogging procedures.

The CSFA process results in a single-pass process that reduces the overall number of steps, displayed in Figure 3.

## 3. Robotic Path Planning

For robotic arm platforms, sections of the surface may lie out of reach, or a given motion may be impossible to execute due to a kinematic singularity [34]. These issues are incurred by a break in the correspondence between Cartesian space and the robot’s fundamental coordinates, the possible joint-positions and link structure. In overcoming the spatial limitations of the robotic manipulator, oriented target-points were converted to configuration space coordinates. As a proof of concept investigation for the deployment of the novel CSFA, test pieces were chosen to test the algorithm’s ability to ensure full coverage on curved and complex surfaces while minimising the risk of collision. Collision avoidance in the test cases were achieved by placing a motion-length limit. To maintain full coverage in the case of required back-tracking, any motion above this joint-space limit would cause the robot to move safely through a known point above the part. In the case of a convex part, point-to-point motion was considered admissible within one step if the subsequent point did not require motion in the current point’s normal direction of more than the sensor-surface stand off. Since the algorithm requires an initial position to be defined along the surface, an initial configuration is given at the start. The path-planner then proceeded to choose the next in Cartesian space, and selected the candidate robotic configuration with the smallest joint-motion. If the selected point induced a configuration motion larger than the allowed threshold, the point was pushed back into the Open-List and another chosen until a suitable point was found or only large-motions were possible. In the latter case, the point with the smallest joint-wise motion was chosen. The robot was then sent joint-wise position command motions, avoiding kinematic singularities and ensuring the reachability of target points.

## 4. Results

Tests on shapes with key non-linear aspects have demonstrated the method’s total coverage of generalised locally differentiable surfaces. The shapes chosen have been selected on the basis of surface irregularities that present challenges to full scanning. Surfaces with cut-outs that are not captured by a global surface spline representation demonstrated the advantage of the algorithm in handling machined parts, or in piece-wise spline produced parts. These are not handled by the nearest available algorithm. Additionally, curved and doubly-curved surfaces were chosen to validate the suitability of the linearisation approximation method. In this section, surfaces chosen demonstrate complete coverage of locally smooth parts and parts with cut-outs. By demonstrating on positive, negative and zero curvature surfaces individually, the iterative and non-recursive algorithm has been validated for all locally smooth and holed surfaces. The process has been implemented in C++, utilising Simon Perrault’s Octree structure [35]. Robotic simulations have been generated using RoboDK software with the Universal-Robotics UR10e as a demonstrative platform, with mesh simulations presented in MeshLab.

**Figure 4:**
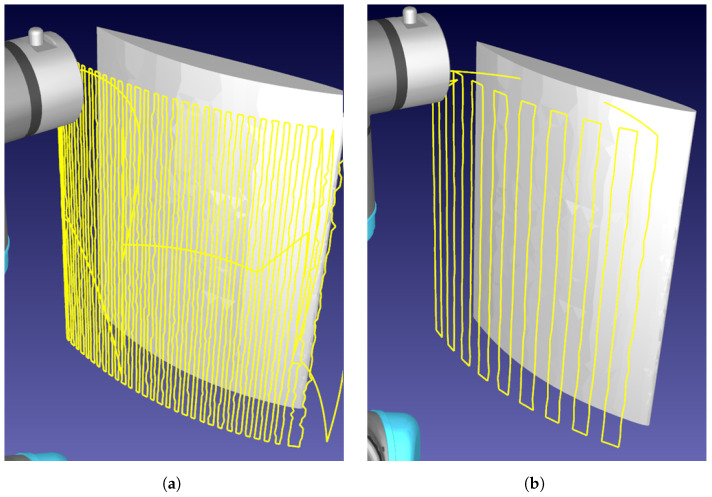
Demonstration of rasterizing a curved aerofoil component. The robotic path is traced in yellow, demonstrating the raster-like path obtained. (**a**) Sampling distance: 3 mm. (**b**) Sampling distance: 10 mm.

The CSFA has demonstrated ease in generating raster-motions on aerofoil components with varying step-sizes, displayed in Figure 4. Due to the relative flatness of the surface, a raster pattern was achieved. For more curved surfaces, there will be over-sampling of the space.

**Figure 5:**
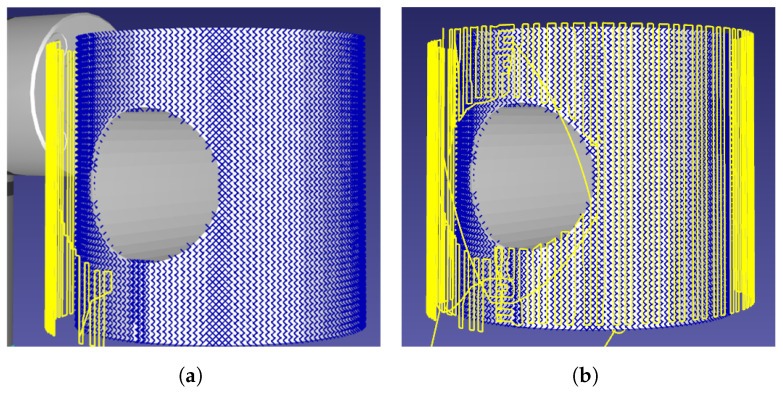
The scan initially misses sections of the pipe due to the shape’s cross-sectional hole.The missed points are picked up at the end of the scan as there is memory of surface-positions to check. Points found are marked in blue, the robotic path traced in yellow. (**a**) Initial scan-pass. (**b**) End-of-scan.

The method has been demonstrated to avoid surface-holes, re-scanning areas previously uncaptured in early-scan stages, displayed in Figure 5. The stack based memory of positions to check allowed effective full-surface discovery in the presence of irregular geometries. Figure 5 demonstrates that the CSFA has a clear advantage over gantry-based delivery platforms, covering complex surfaces without visiting the holed regions while still capturing the whole surface without needing the planar limits of the plate as input.

**Figure 6:**
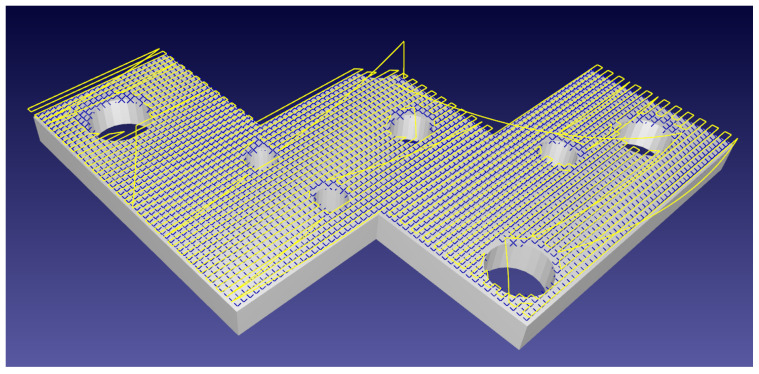
A complex flat plate holed with differently sized voids. The robotic path in yellow backtracks to allow for full surface discovery, shown by blue crosses, in the presence of surface-discontinuities.

Repeatedly holed surfaces present multiple points of return, demonstrated in Figure 6.

**Figure 7:**
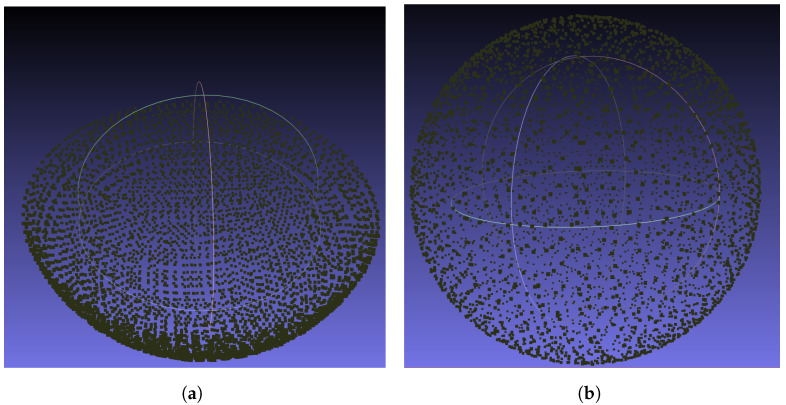
Points discovered while simulating a scan on a bowl and sphere of radius 150 mm with a sampling distance of 3 mm. (**a**) Concave shape sampling. (**b**) Sphere sampling.

The CSFA process makes a linear approximation of the surface in the neighbourhoods of discrete points. Displaying the algorithm on surfaces of positive and negative curvature, as in the sphere and bowl, demonstrates that it is robust in cases of local non-flatness. This is displayed in Figure 7.

**Figure 8:**
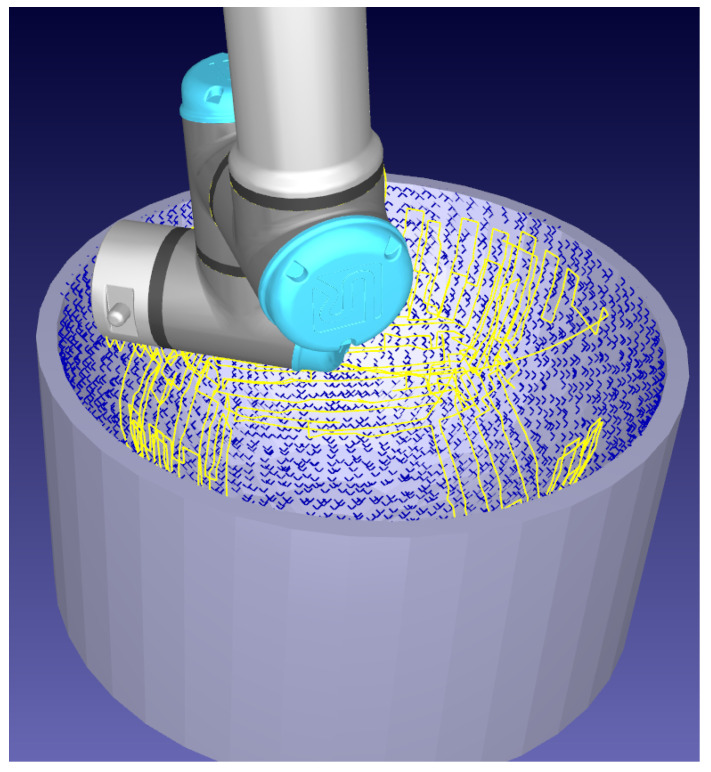
Sampling on a concave shape. The robotic path, that can form irregular patterns without a preferred direction, is shown in yellow. Discovered points on the bowl are shown as blue crosses.

The irregular rasterization pattern may be seen in Figure 8. Unlike for surfaces of only one direction of curvature such as in Figure 4 or Figure 5, rasterization for double-curvature surfaces is irregular. This incurs inefficient motions compared to traditional spiral-rasterization patterns.

**Figure 9:**
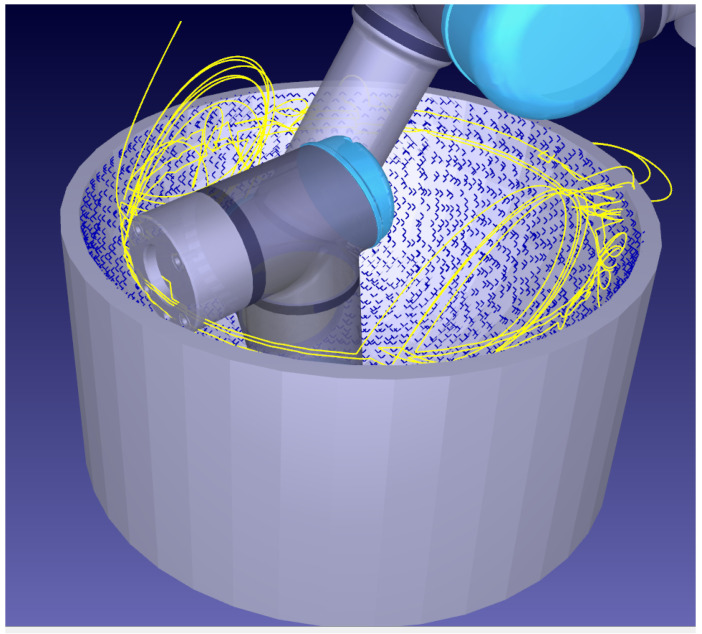
Sub optimal horizontal rasterization of a concave surface. Yellow trace lines demonstrate costly re-arrangement procedures to discover all the points shown in blue.

A horizontal rasterization pattern of subsequent circles resembling traditional spiralized patterns may be imposed by using a preferred direction vector; however, they can result in large re-arrangement procedures seen in Figure 9.

**Figure 10:**
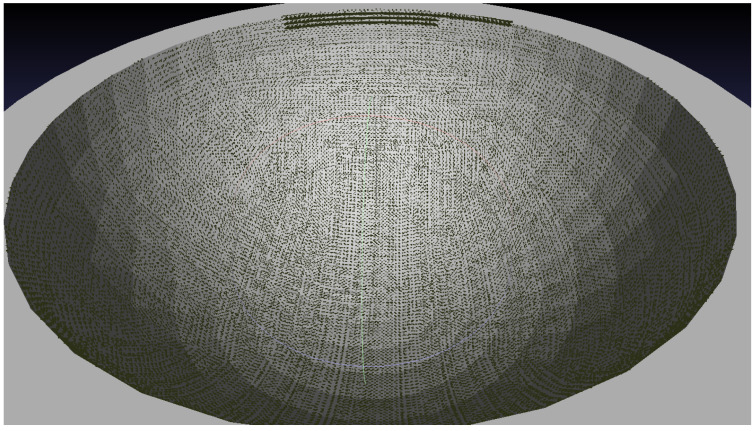
Points in bold display the extent of discovery with no over-sampling regime. Sampling rate: 1 mm, radius of bowl: 150 mm.

Curvature considerations are also demonstrably necessary for full surface coverage of components. Without over-sampling the space based on known surface curvature, full coverage is not guaranteed since taking a step will not necessarily take the algorithm to a new Octree-leaf. In turn, the algorithm stops prematurely as it aliases the points before and after the step within the Octree map. The effect of this is displayed in Figure 10.

## 5. Experimental Results

Complete coverage of locally differentiable surfaces has been shown in simulation when there are no limitations due to the robotic platform or sensor. Two key test pieces were identified to validate the algorithm’s practicality in deployment. These were a surface of doubled-curvature and a surface with a cut-out. The doubly curved surface has been chosen to show that with the correct step size, sensors with small ranges may complete the search process, and that the approximation found for the surface normal is a suitable one. Moreover, since the important quantity in Octree sampling to guarantee completeness is the ratio of curvature to step size, the doubly curved surface shows that the heuristic presented is applicable to surfaces of all curvatures, given a step size that does not hinder sensor-surface coupling. The part with a section cut out further validates the approach when the surface is not globally represented by a global b-spline, as is necessary within the nearest algorithm. Since the algorithm utilises an iterative and non recursive heuristic, by demonstrating the process on these surfaces it is also demonstrated to work on curved surfaces with varying curvature and with cut-outs. It is important to note that the hardware chosen for completing the scanning process is the limiting factor, as smaller sensors are necessary to complete scans on objects that have extreme curvatures.

**Figure 11:**
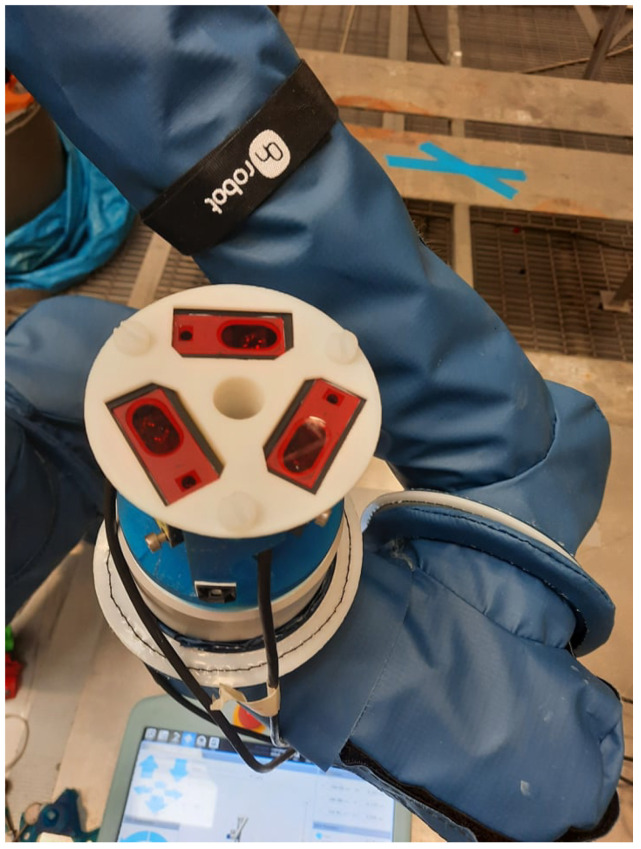
The tri-laser holder, attached to the UR10e flange. The design with rotational symmetry around axis 6 of the robot minimised the footprint of the tool.

Experimental testing of the CSFA utilised three flange-mounted Panasonic HG-C1030-P lasers, connected to an Arduino board for real-time data collection. The laser’s viewing range was 30 mm ± 5 mm, limiting the feasible step size over highly curved surfaces, as height variations of over 5 mm over the step would remove the possibility of further surface discovery. The laser’s repeatability did not affect motion planning, as it was in the range of 10 μm. The lasers were held within a 3D-printed cradle displayed in Figure 11. An external laptop collected data from the Arduino and Universal-Robots UR10e robotic platform simultaneously. Connecting through a COM port and Ethernet-enabled TCP/IP connection, respectively, position data and commands were received and sent to the robot. The CSFA, data interpretation, and inverse kinematics solutions were coded in C++. The external laptop had an Intel Core i5 processor with the program built and run from a Visual Studio programming environment. Results were imaged using Meshlab.

**Figure 12:**
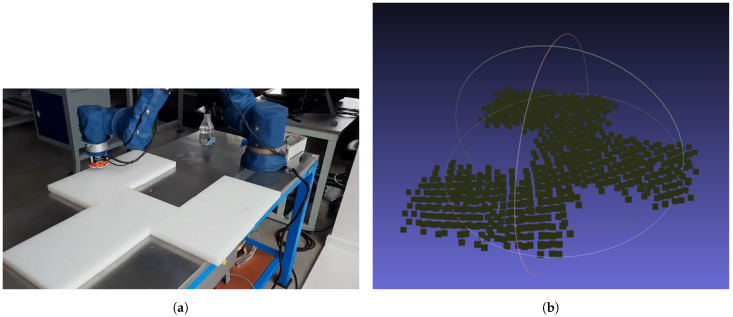
Automatic online profiling and scanning of an object with non-smooth shape. After a new point is found, the UT probe is applied to collect data. (**a**) Non-smooth shape created from arranged plates. (**b**) Resultant point cloud collected by the tri-laser and projected to the World-Frame using the live Joint-position of the robot.

To represent a non globally smooth b-splineable surface, laminate plates were placed into a planar pattern with a cut out displayed in Figure 12a alongside the point-cloud of collected data displayed in Figure 12b. Full discovery of the target surface demonstrates the applicability of the CSFA in cases where a direct path along the surface to every point is not possible. The recollection of hypothesised points to visit allows traversal around corners, completely scanning regions with no direct path to one another.

**Figure 13:**
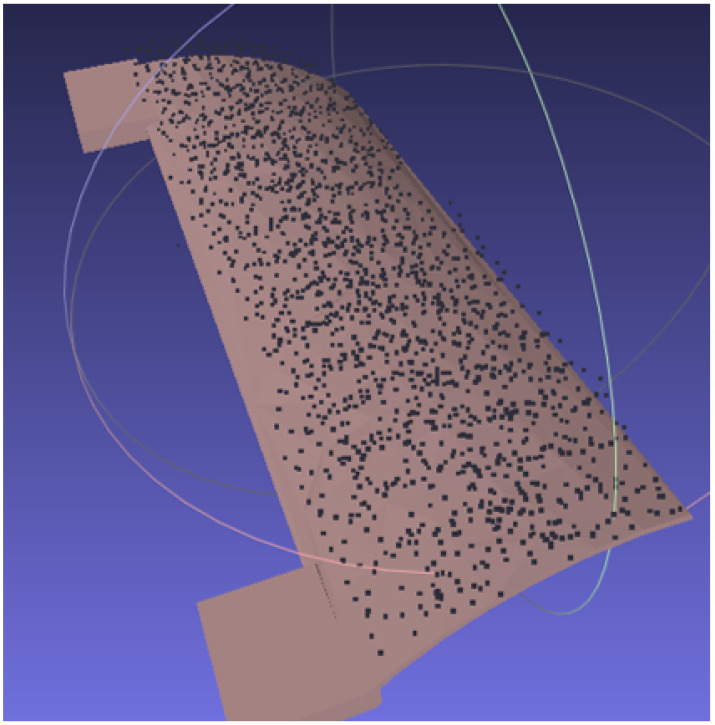
Point Cloud of a complex doubly-curved surface profiled in real time, aligned to the CAD model in post-processing.

A curved mock-aerofoil segment provided additional experimental data displaying application to a use-case commonly seen within NDT in Figure 13. The total time taken for this use-case was 7 min 30 s for 3 cm spaced collection points. Providing a real-world use-case for NDT, the full surface discovery of a doubly-curved surface with no-prior path planning provides the proof of concept for single-pass profiling of a complex surface and validation for the linearised surface approximation, while the part is relatively small compared to the robot’s reach, the strength of this example is in the surface’s extreme curvature. This use-case validates the application to surfaces commonly seen as complex within NDT.

**Figure 14:**
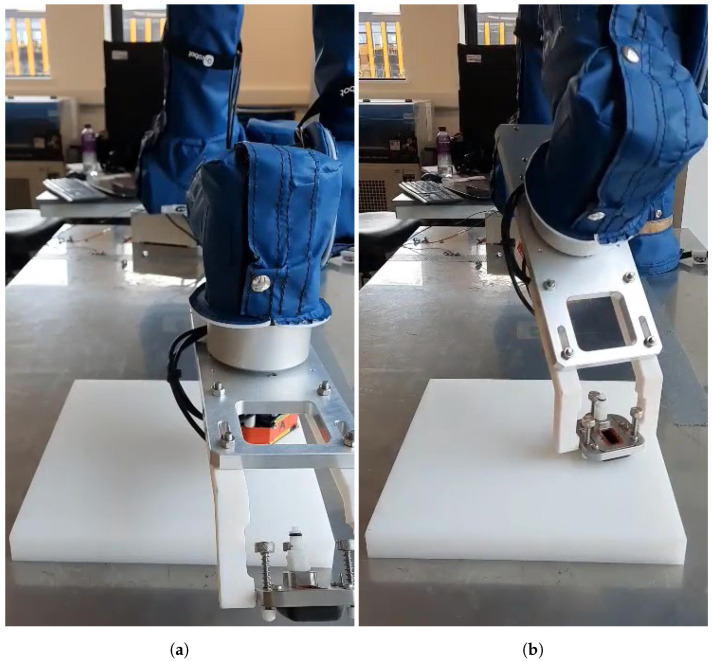
Automatic online single-pass profiling of a surface. (**a**) Initial non-contact surface discovery and profiling with the tri-laser. (**b**) Subsequent application of the Conformable-Wedge coupled UT device.

Finally, the proof of concept for simultaneous non-contact surface profiling with the tri-laser platform combined with Conformable-Wedge-Probe scanning is presented. The process is two-step; the tri-laser discovers the surface, displayed in Figure 14a, the tool reversed and the Conformable Wedge Probe applied to the discovered position, displayed in Figure 14b.

In deployment, sensor ranges provided the most significant challenge. Since the tool’s base had a diameter of 5 cm, the curvature of parts observed within that region had to not exceed the viewing range of the laser-sensors in order to ensure the tool and part did not collide.

The main source of risk to deployment was an incorrect laser-tool calibration. During early testing, the sensor’s beam had an orientation offset that with larger step-sizes often risking collisions with the part. Scanning the planar part with a re-printed tool that corrected the laser-flange alignment, and calibrated using the four-point method, the standard deviation of points from the horizontal plane was 0.81 mm with mean signed-error of $O\;\left(10^{-16}\;\mathrm{mm}\right)$.

Further, while demonstrations were limited by the lack of a collision avoidance schema, these experiments have proven the algorithm’s capability in autonomous scanning processes, and applicability to robotic NDT. The main challenge facing industrial deployment of robotic NDT where parts have no accurate digital-twin is the flexibility of the robotic platforms in use, and their ability to define complete surface coverage. We have proven the ability of this algorithm to overcome this issue in realistic contexts.

## 6. Discussion

The authors have successfully implemented an adaptation of the FFA for full coverage of free form surfaces. The implementation has been demonstrated on positive and negative curvature surfaces, highlighting how the linearised approximation is not a detriment to overall surface following capabilities of the algorithm.

In post-processing, the CSFA has been shown to output a raster-path along arbitrarily locally differentiable surfaces. For doubly-curved surfaces, the rasterization pattern becomes irregular and there is an over-sampling of points. However, the method ensures total coverage of the part which is preferable in NDT to sparse sampling. The potential applications of the algorithm are not limited to automatic rasterization procedures. The Octree memory method would allow fully automated discovery and scanning of structures with any robotic platform, such as mobile robots traversing a large structure. Further, the traversal method can be applied with any limited-aperture sensor, enabling a generalised surface-movement strategy when sensor data is limited. Finally, the discrete-point approach allows the method to capture surfaces that cannot be globally splined. The limitation in the case of significant surface discontinuities such as part-edges is that the process will not necessarily find the other side of the part, discovery determined by the sensor’s range and aperture size relative to the discontinuity. In practical deployments the sensor range was the key limitation, limiting the sensors step size due to the surface curvature so as to continue full surface discovery. Practical demonstrations applied to complex cut-out surfaces and realistic doubly curved aerofoil mock-ups show the real-world application with limited-range laser sensors. Proof of concept for wedge-probe coupled UT applications provide the NDT specific aims of this paper of removing the need to path plan for full-surface scanning.

For complex surfaces such as aerofoils or machined plates with cut-outs, the algorithm demonstrated is safe for deployment. For more complex shapes such as external pipe-scans, limited knowledge of the environment is necessary to prevent collisions. Future work will deploy the algorithm using low-cost environmental sensors to prevent collisions and path planning such as Rapidly exploring Random Trees (RRT) algorithms to scan complex components.

Future works investigating online surface profiling will further consider options to remove the necessity for user-inputted curvature estimates and step-sizes entirely. Adaptations to specific sensor types for surface profiling shall also be considered.

## Data Availability

Not applicable.
